# Young Children’s Sensitivity to Their Own Ignorance in Informing Others

**DOI:** 10.1371/journal.pone.0152595

**Published:** 2016-03-29

**Authors:** Sunae Kim, Markus Paulus, Beate Sodian, Joelle Proust

**Affiliations:** 1 Ludwig Maximilian University of Munich, Munich, Germany; 2 Sabanci University, Istanbul, Turkey; 3 École Normale Supérieure, Paris, France; CNR, ITALY

## Abstract

Prior research suggests that young children selectively inform others depending on others’ knowledge states. Yet, little is known whether children selectively inform others depending on their own knowledge states. To explore this issue, we manipulated 3- to 4-year-old children’s knowledge about the content of a box and assessed the impact on their decisions to inform another person. Moreover, we assessed the presence of uncertainty gestures while they inform another person in light of the suggestions that children's gestures reflect early developing, perhaps transient, epistemic sensitivity. Finally, we compared children’s performance in the informing context to their explicit verbal judgment of their knowledge states to further confirm the existence of a performance gap between the two tasks. In their decisions to inform, children tend to accurately assess their ignorance, whereas they tend to overestimate their own knowledge states when asked to explicitly report them. Moreover, children display different levels of uncertainty gestures depending on the varying degrees of their informational access. These findings suggest that children’s implicit awareness of their own ignorance may be facilitated in a social, communicative context.

## Introduction

Understanding one’s own knowledge states is critical for learning as well as for social interaction, including conversation. One such case is when we inform others. We should refrain from informing others when we do not know as opposed to when we do know. It is not clear, however, whether children inform others selectively depending on the validity and informative value of their own knowledge states. Children may be helpful and reliable informants of others, but they might also be overconfident in their own ability to inform. By drawing on the metacognitive literature, we explored whether children are sensitive to their own ignorance when they are asked to inform another ignorant person. Below, we review research studies on children’s informing and gestures and then turn to a body of research on implicit and explicit forms of metacognition.

A few research studies suggest that children at an early age selectively inform their conversational partners depending on their attribution of knowledge states to others [1–2]. Even 12-month-old infants display sensitivity to others’ knowledge states in their informing behavior [3]. Thus, children inform an ignorant person rather than a knowledgeable person. Yet, this line of research has exclusively focused on children’s sensitivity to others’ knowledge states when informing them. Being an efficient informer, however, also requires sensitivity to one’s own knowledge state. For example, if we were ignorant about which way to take to the cinema, it would not be useful to inform a help-seeking stranger on the street by giving him a direction. Therefore, a full-fledged form of selective informing should rest upon considering both others’ knowledge states and one’s own.

Although surprisingly little research has addressed this issue, we have good reasons to assume that already by preschool age children take their own knowledge states into account when informing others. Toddlers show some nascent awareness of their lack of knowledge in communication ([4] see also [5, 6]). Moreover, it is often assumed that children’s understanding of others’ and of their own knowledge states are closely related to one another, because the same conceptual resources should be used in both cases (e.g., [7] but see also [8]). Research shows, however, that both forms of understanding might crucially depend on nonconceptual resources. For example, preschoolers’ epistemic sensitivity to what others know or do not know is modulated by activity-dependent cues such as fluency (i.e., how easy one can process another person’s communicative message) [9]. Taken together, preschool children’s informing decisions may be modulated by a sensitivity to their own knowledge states.

A separate domain of research on young children’s metacognitive abilities provides an insight into whether children are sensitive to their own knowledge states. Studies show that by 4 years of age children accurately report their own knowledge or ignorance when they are allowed vs. denied full access to relevant perceptual information (e.g.,[10–11]). However, in a ‘partial exposure’ task in which children are shown multiple objects but do not know which particular object is then hidden in a container, an accurate assessment of their own knowledge states is a late developmental achievement [12–13]. For example, in a recent study by Rohwer et al. [13], children were presented with two toys and were told that only one of the toys would be hidden in a box. After the hiding of one of the toys, children were asked if they did or did not know the contents of the box. Children younger than 6 years of age tended to overestimate their knowledge by responding that they knew the hidden object in the box.

Based on their findings, Rowher et al. [13], argue that a fully *representational* understanding of the causal origin of one’s own knowledge is not acquired until around the age of 6 years. Supporting this view, some older studies showed that preschool-aged children often inaccurately reported that they had always known the information they had just learned or misidentified the specific modality of the senses (seeing vs. touching) required for knowing a certain piece of knowledge (e.g., [14–15]).

However, metacognition does not necessarily involve explicit awareness of mental and cognitive processes [16–17]. Moreover, using tasks that do not require explicit verbal responses, recent empirical studies document implicit metacognitive abilities in preschool aged children and infants [18–22] and even nonhuman animals ([23–24] see [25] for a review). One such task uses an ‘opt-out’ paradigm in which young children and nonhuman animals are allowed to skip trials (e.g., [18, 19, 21, 23, 26]). Participants tend to use the option of skipping trials when they are uncertain about their responses, thereby avoiding inaccurate responses. Therefore, young children may have an implicit ability to monitor their own mental states without being able to explicitly verbalize them. In particular, informing decisions—whether to inform or not—as discussed earlier can be a good index for children’s emerging sensitivity to their own epistemic (un)certainty.

In addition to children’s informing decisions, their spontaneous gestures might also give valuable information on their sensitivity to their own knowledge states. Research by Goldin-Meadow and colleagues has shown that children’s manual or pointing gestures reveal an earlier understanding than their verbal judgments (e.g.,[27–28]). For example, in a Piagetian conservation task, children tend to consider only one factor (the height of a glass containing water) while ignoring another relevant factor (the width of the glass) in their verbal judgments whereas their gestures indicate they do consider both factors [27]. Thus, the gestures that children produce can reveal their early developing implicit- and transitional- knowledge.

Interestingly, adults produce gestures that mark their epistemic stance and these gestures are perceived by listeners as conveying the epistemic stance of the speaker [29]. Thus, epistemic gestures seem to be a reliable index of the speakers’ epistemic states in communication. Additionally, although not as frequent or accurate as adults, 7- and 8-year-old children produce uncertainty related audio-visual cues (e.g., ‘funny faces,’ fillers, eyebrow movement) and are able to detect them displayed by others [30]. By implication, young children might spontaneously—and perhaps unknowingly—produce uncertainty gestures even if they do not explicitly mark their uncertainty in their verbal communication.

In the present research, we manipulated children’s epistemic access to the contents of a box and asked whether 3- and 4-year-old children display sensitivity to their own ignorance when they are asked to inform another ignorant person. Importantly, by adopting the opt-out paradigm used in prior studies of metacognitive abilities in nonhuman animals and young children (e.g.,[18, 19, 21, 23, 26]), we gave children an option to decline informing. Moreover, in order to assess whether children’s spontaneous gesture production indicates their varying level of uncertainty, we also investigated children’s uncertainty gestures. Finally, in order to investigate the gap between explicit and implicit forms of metacognition documented in the prior studies, in addition to an ‘Informing task’ in which children were asked to inform another person, all children received an ‘Explicit task’. Here, we relied on the task by Rohwer et al. [13] in which participants were asked to verbally report their knowledge states (see Method section below). We expected that children would selectively inform others depending on their knowledge states by agreeing to inform others when they are more certain about their knowledge states. By contrast, children would over-estimate their knowledge states in their explicit verbal judgments as in Rowher et al. [13]. Given the prior evidence showing that children as young as 3 years old (if not younger) display some early metacognitive abilities (e.g.,[18, 19, 21, 23]) and that they are good conversational partners (e.g., [4]), we expected that both 3-and 4-year-old children would show sensitivity to their own ignorance in their informing in the present tasks. We also explored possible age effects because some prior studies suggest that children’s metacognitive abilities—despite its early presence in development- develop during preschool years (e.g., [9, 31]).

## Method

### Participants

Three-year-old (N = 18, Mean age = 42 months 15 days, Range = 38 months 24 days ~ 45 months 6 days, 7 boys, 11 girls) and 4-year-old German children (N = 18, Mean age = 55 months 7 days, Range = 48 months 11 days ~ 56 months 18 days, 10 boys, 8 girls) participated. Five additional 3-year-old children were tested but excluded from final data analyses (1 child due to experimenter error; 1 child who inaccurately labeled the toys at the end of the testing; 1 child due to a language delay; 2 children who did not provide any responses throughout the entire experimental session). Children were largely from middle or upper middle class families. Parental written consent forms were obtained on behalf of the children. Children were asked if they would be willing to participate before the testing began and told that they can stop at any point. This study, including the consent procedure, was approved by the Ethics Board of Ludwig Maximilian University of Munich, Germany.

### Materials

**Fig 1:**
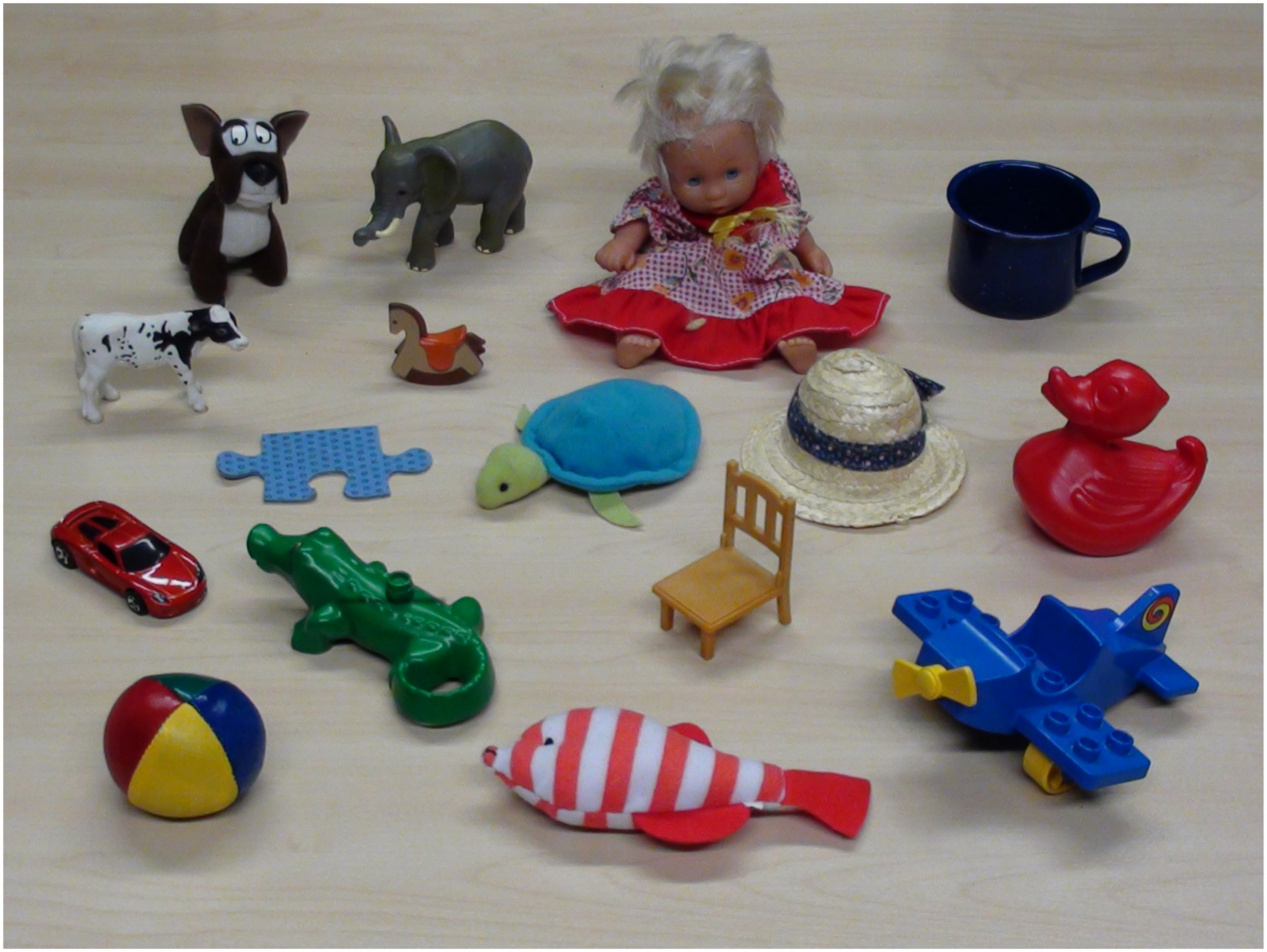
A photo of toys. Toys used in the experiment.

A white partition wall (50 · 50 · 1 cm) was used as a screen to restrict children’s view. In the informing task, two additional white partition walls were used, one between the experimenter and the child, and the other between another adult and the child. We used a black, opaque shoebox with a lid as a hiding place for the toys. Two sets of 8 different toys were used for the two tasks (see Fig 1).

### Design and procedure

Children were tested in a laboratory room, seated at a table opposite to the experimenter. Each child received an “informing task” followed by an “explicit task.” Children also participated in a short filler activity (watching a movie) between the two tasks (about 5 minutes). For each task, children completed three conditions (Ignorance, Partial Knowledge, Full Knowledge), two trials per condition, in a counterbalanced order. The order of the trials in the informing task was repeated for the explicit task.

#### Informing task

**Fig 2:**
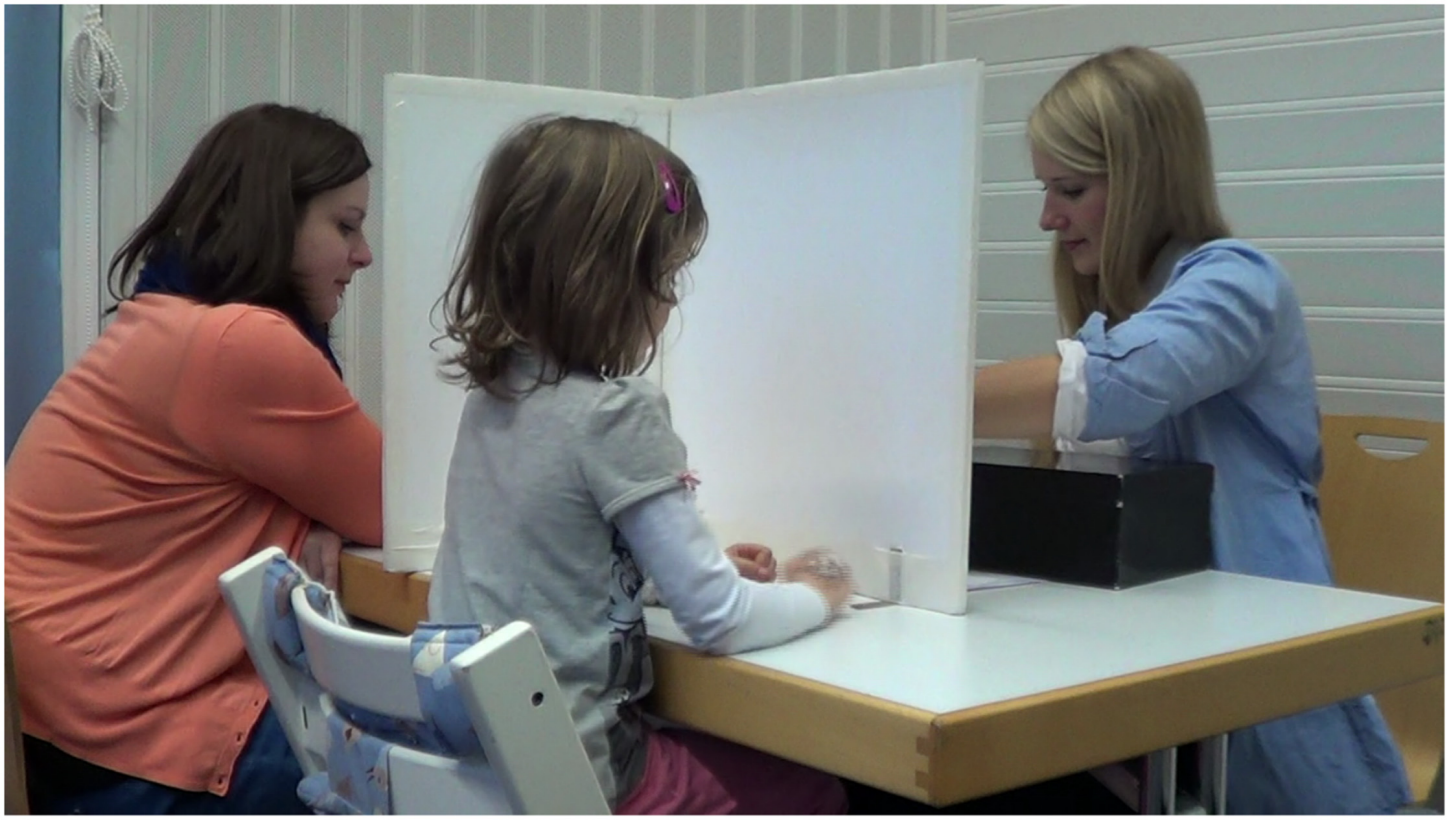
A photo of the informing task. The positions of the experimenter, a child participant, and Max in the informing task.

We adopted a modified procedure from Rohwer and colleagues [13]. Another adult (“Max”) was seated next to the individual child and was present during the entire informing task session. There were three separate removable screens—one between Max and the child, another between the child and the experimenter, and the third between Max and the experimenter. Max’s view of the box was blocked (see Fig 2).

In the Ignorance condition, children were not presented with any objects and told that a toy would be placed in the box (“I’m going to put a toy inside the box”). Then, behind the screen, the experimenter put a toy in the box, removed the screen, and told the children, “Now I have put a toy inside the box.” In the Partial Knowledge condition, children were presented with two different toys and the empty box and told that one of the toys would be placed inside the box (“I‘m going to put one of these toys inside the box”). After hiding one of the toys in the box behind the screen and removing the screen, the experimenter told, “Now I have put one of the toys inside the box.” In the Full Knowledge condition, children were presented with a toy and the empty box and told that the toy would be placed inside the box (“I’m going to put this toy inside the box”). After placing the object inside the box, closing the box with a lid, the experimenter told the children, “Now I have put the toy inside the box.” In all conditions, instead of being asked to explicitly indicate their own knowledge states as in Rohwer et al. [13], children were asked if they were willing to inform another ignorant person, “Max wants to know what’s inside the box. Can you help him? If you do not want to tell him, it’s okay. I can tell him.” If children agreed to inform, then the experimenter removed the screen between the child and Max, and said, “Go ahead and tell him.” If children declined to inform, then the experimenter informed Max after removing the screen between herself and Max, “Okay, I will tell him. There is a [ball] inside.” Then, the screen was placed back on the table.

#### Explicit task

The explicit task closely followed the procedure of Rohwer and colleagues [13]. The procedure was the same as in the informing task except for the following changes. There was no third person children are asked to inform. In all conditions, children were explicitly asked to state their knowledge states, “Do you know what’s inside the box or do you not know?” In all three conditions, follow-up questions depended on children’s responses. If children said that they did not know the content of the box, they were asked, “Why don’t you know what’s inside the box?” If children said that they did know the content of the box, they were asked the following two questions: 1) “Okay, then tell me what’s inside the box?” 2) Do you really know or are you just guessing?”

At the end of the explicit task, children were asked to label the toys used in the two tasks. All children were able to label them except for one child whose responses were excluded from the final data analyses.

We coded the following gestures during the time (about 5 seconds) when children were asked whether or not they would inform Max in the informing task and whether or not they knew the contents of the box in the explicit task: tilting the head to one side; shaking the head; shrugging shoulders; looking away. The experimenter and a second coder (blind to hypotheses) independently coded all the data. Interrater reliability was 100%.

## Results

### Children’s informing decision

**Fig 3:**
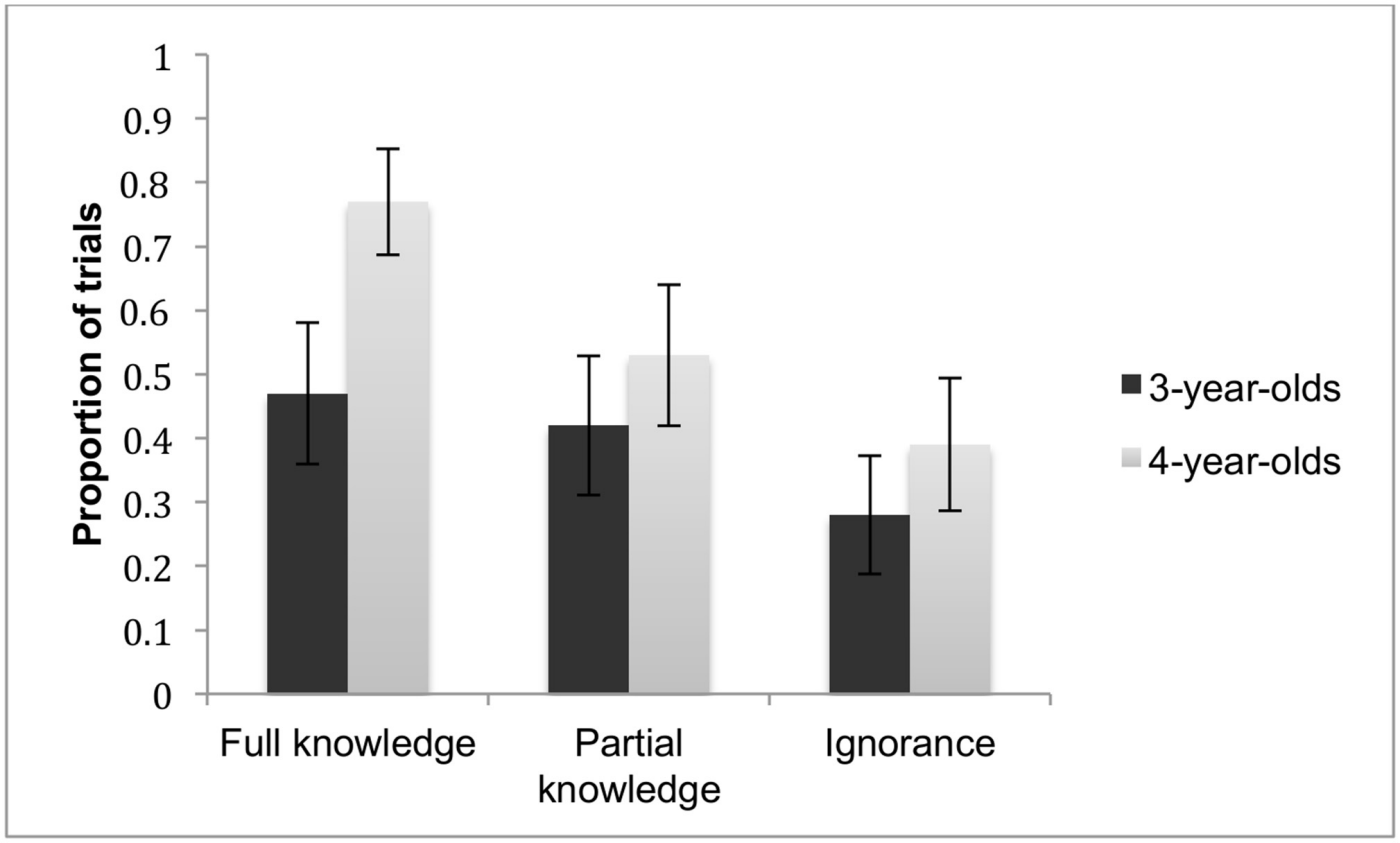
Informing decisions. The mean proportion of trials in which children chose to inform (as opposed to declining to inform). Error bars indicate standard error.

We first asked whether children’s performance in the informing task reveals sensitivity to their differing knowledge states across conditions. Thus, we asked whether children’s initial decision to inform (agreeing vs. declining) varied by their knowledge states. Children were given a score of 1 if they agreed to inform and a score of 0 if they declined to inform. Fig 3 presents the mean proportion of trials in which children agreed to inform.

These scores were analyzed by means of a 2 (Age: 3-year-olds vs. 4-year-olds) by 3 (Condition: Full knowledge, Partial knowledge, Ignorance) repeated ANOVA with Age as a between-subject factor and Condition as a within-subject factor. There was a significant main effect of Condition, *F* (2, 68) = 9.35, *p* < .001, *η*^*2*^ = .22 (Full knowledge: *M* = .63 *SD* = .07; Partial knowledge: *M* = .47 *SD* = .08; Ignorance: *M* = .33 *SD* = .07). More specifically, children’s decision to inform decreased in a linear fashion across the three conditions, *F* (1, 68) = 14.17, *p* < .01. The interaction effect was not significant, *F* (2, 68) = 1.38, nor was the effect of Age, *F* (1, 34) = 2.10.

### Uncertainty gesture

**Fig 4:**
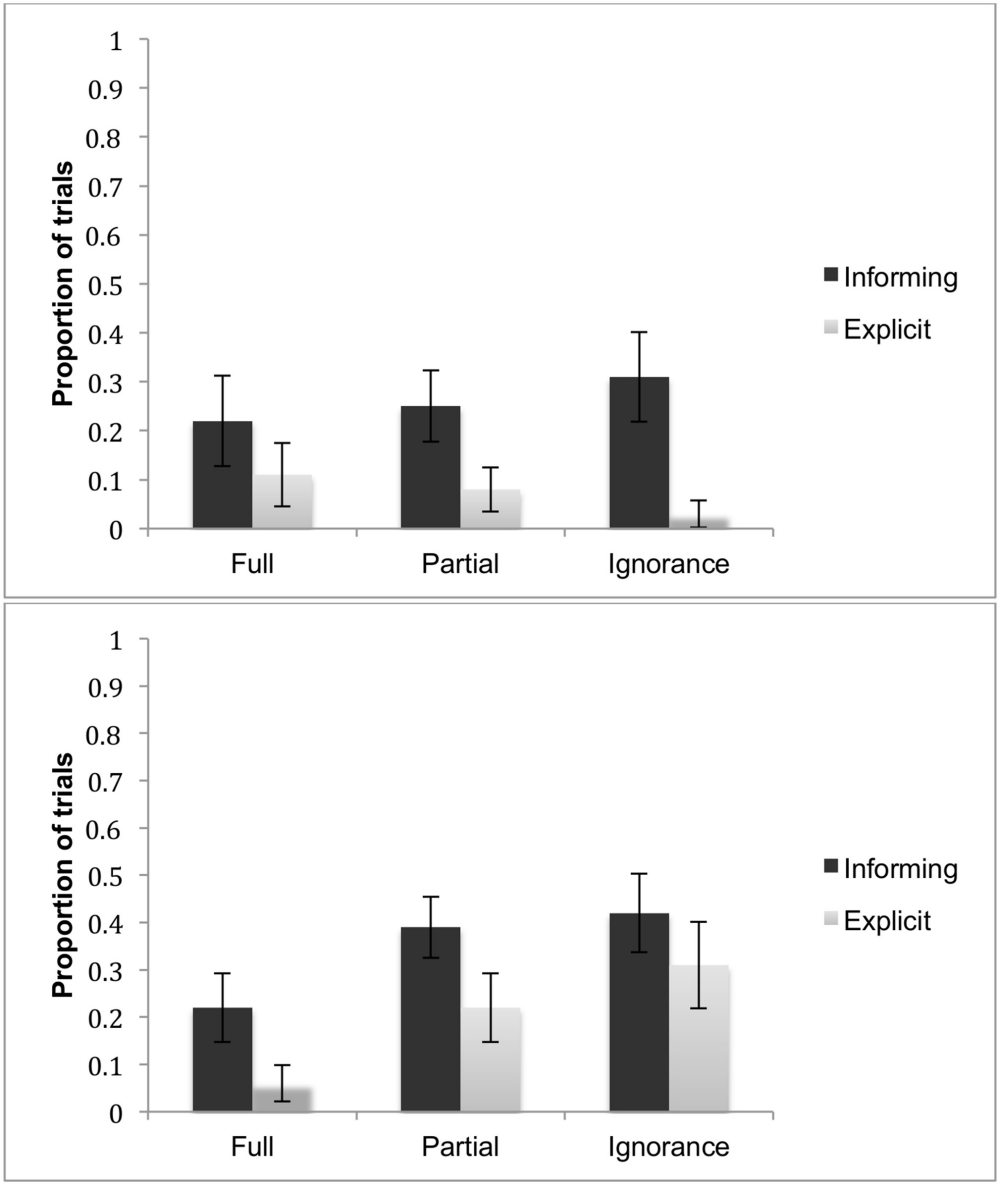
Uncertainty gestures. The mean proportion of trials in which children’s uncertainty gestures were present as a function of Age (3-year-olds: upper panel; 4-year-olds: lower panel), Condition and Task type. Error bars indicate standard error.

Next, we asked whether children’s uncertainty gesture production varied with condition and task type. Fig 4 presents the mean proportion of trials in which an uncertainty gesture was present.

The data were analyzed by means of a 2 (Age: 3-year-olds vs. 4-year-olds) by 2 (Task type: informing vs. explicit) by 3 (Condition: Full knowledge vs. Partial knowledge vs. Ignorance) ANOVA with Age as a between-subject factor and Task type and Condition as within-subject factors. Uncertainty gestures were produced more frequently in the informing task than in the explicit task, *F*(1, 34) = 15.78, *p* < .001, *η*^*2*^ = .32. Moreover, the production of the uncertainty gestures differed by condition, *F*(2, 68) = 5.70, *p* < .01, *η*^*2*^ = .14: production increased linearly across the three conditions, *F* (1, 34) = 9.38, *p* < .01. An interaction of Condition X Age was also significant, *F*(2, 68) = 5.70, *p* < .01, *η*^*2*^ = .14. The simple effect of Age was significant only for the Ignorance condition (p < .05): 4-year-olds as compared to 3-year-olds produced more uncertainty gestures in the Ignorance condition. The simple effect of Condition was significant only among 4-year-olds. More specifically, 4-year-olds’ uncertainty gesture production was less frequent in the Full knowledge condition as compared to the Partial knowledge condition or the Ignorance condition (both *ps* < .001), but it was not significantly different between the Partial knowledge and the Ignorance condition (*p* > .05). Three-year-olds did not differ in their uncertainty gesture production across conditions (all ps > .05). None of the other effects were significant.

### Comparison between the Informing and Explicit tasks

Finally, we compared children’s sensitivity to their own ignorance in the informing task to that in the explicit task in order to test the gap between the two tasks. We recoded the data in the following way. The coding in the explicit task followed that of Rohwer et al. [13[. In the Full Knowledge condition, children received a score of 1 if they correctly reported their own knowledge, and a score of 0 if they did not. In the Ignorance condition and the Partial Knowledge condition, children received a score of 1 if they acknowledged their ignorance in response to the test question (“Do you know or do you not know what’s inside the box?”). Children also received a score of 1 if they erroneously indicated an object name to the test question, but acknowledged that they were guessing when asked the follow-up question. In the informing task, in the Full knowledge condition, children received a score of 1 if they agreed to inform and accurately reported the object identity to Max and a score of 0 if they declined to inform. In the Partial Knowledge and Ignorance conditions, children received a score of 1 if they declined to inform or if they agreed to inform but indicated their uncertainty or ignorance about object identities to Max (e.g., “Hmm,” “I don’t know”). We also coded those responses in the partial knowledge condition that indicated two possible objects (e.g., “a ball or a car”) as correct. These responses constituted only a small portion of the data and the results remain the same if they are excluded.

**Fig 5:**
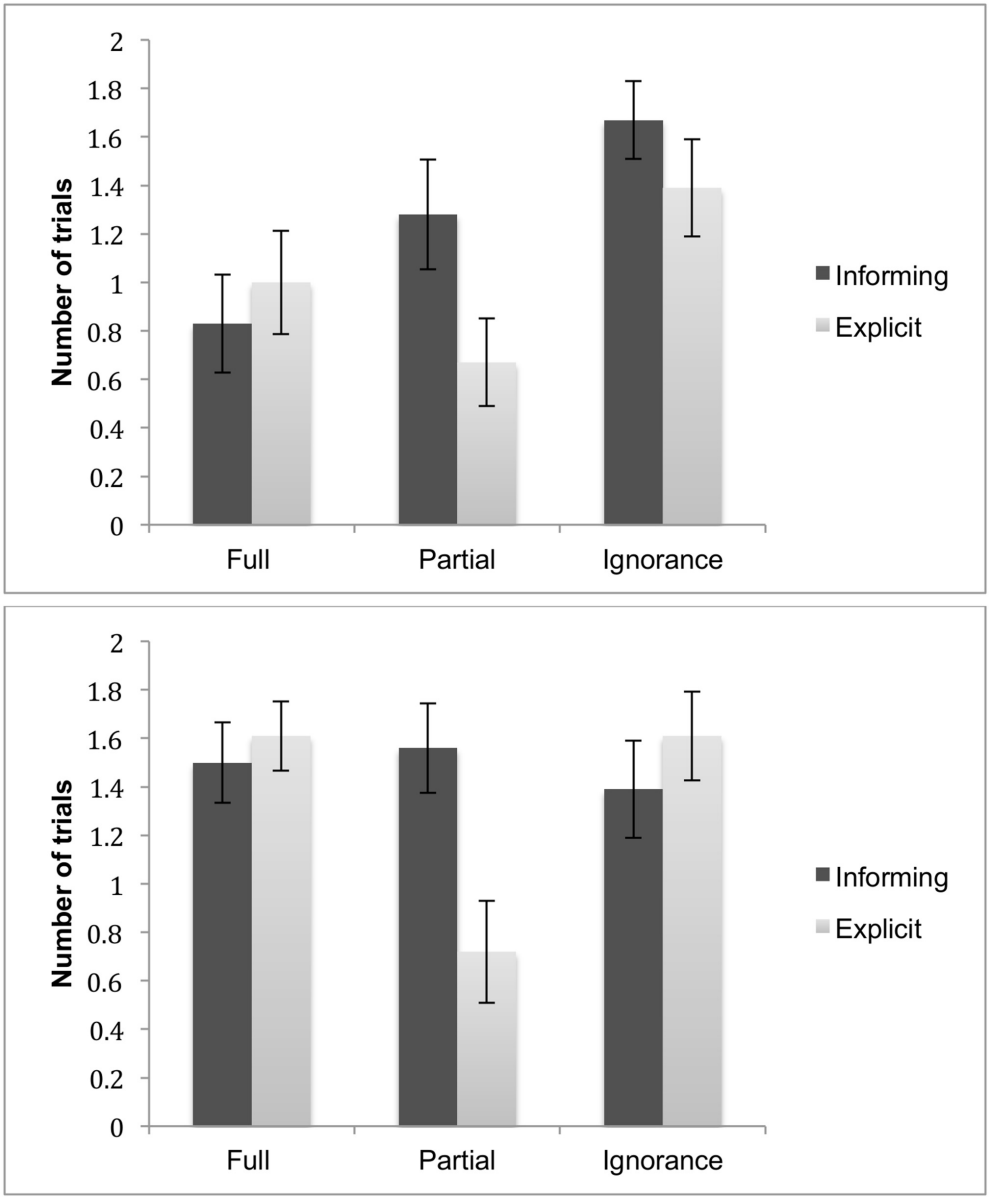
Informing and explicit tasks. The mean number of trials in which children provided accurate responses as a function of Age (3-year-olds: upper panel; 4-year-olds: lower panel), Condition and Task type. Error bars indicate standard error.

Fig 5 presents the children’s mean scores as a function of Age, Condition and Task types.

These responses were analyzed with a 2 (Age: 3-year-olds vs. 4-year-olds) by 2 (Task type: explicit vs. informing) by 3 (Condition: Full knowledge, Partial knowledge, Ignorance) repeated measures ANOVA with Age as a between-subject factor and Task type and Condition as within-subject factors. There was a significant main effect of Age *F*(1, 34) = 5.34, *p* < .05. *η*^*2*^ = .14. Four-year-olds were more accurate in assessing their knowledge than 3-year-olds. There was also a significant main effect of Task type *F*(1, 34) = 5.44, *p* < .05. *η*^*2*^ = .14, a significant main effect of Condition *F* (2, 68) = 4.16, *p* < .05. *η*^*2*^ = .11, and a significant interaction of Task type X Condition, *F*(2, 68) = 7.54, *p* < .01. *η*^*2*^ = .18.

Inspection of Fig 5 shows that both 3-year-olds (upper panel) and 4-year-olds (lower panel) performed relatively poorly in the partial knowledge condition but only in the explicit task. To check these conclusions, the interaction effect was further analyzed by conducting tests of simple effects. The simple effect of Task type was significant only for the Partial knowledge condition (*p* < .0001). Children’s performance did not significantly differ between the two tasks in either the Full knowledge condition or the Ignorance condition (both *p*s > .05). For the explicit task, the simple effect test of Condition revealed that children performed significantly worse in the Partial Knowledge condition than in the Full knowledge condition (*p* < .01) or the Ignorance condition (*p* < .0001). For the informing task, the simple effect of Condition was not significant for any of the pair-wise comparisons. None of the other main effects or interaction effects were significant.

## Discussion

Young children displayed sensitivity to their own ignorance in their decisions of informing others. Interestingly, whereas they over-reported their knowledge in the explicit verbal judgment task, when they were asked to inform another person they were more accurate in conveying their ignorance either by declining to inform or by producing gestures of uncertainty. Below, we discuss the implications of these findings.

The present findings extend prior research on children’s early communication skills. A few studies have suggested that infants and young children selectively inform another person depending on the person’s knowledge states ([1–3] but see [32]). However, all these studies focus on whether children take *others*' knowledge states into account when informing them. The current study expands this line of research by demonstrating that children also selectively inform others depending on their *own* knowledge states. Children’s propensity to inform others is modulated by their own (un)certainty.

According to Harris [5] and Lohmann and Tomasello [6], communication can promote toddlers’ understanding of their conversational partners as epistemic beings (see also [33]). Likewise, conversation could allow children to become more gradually aware of their own, variable, epistemic states. Although the present research did not involve a richly interactive context, it is consistent with theoretical proposals that young children’s sensitivity to their own ignorance may be facilitated in the context of such dialogic communication.

The present findings also provide some novel information to a domain of research on young children’s metacognitive abilities. By age 4, children are sensitive to the relationship between perceptual access and knowledge states when they are either given or denied *full* access to information (e.g., [10]), but they fail to manifest such sensitivity when given *partial* access to information (e.g., [13]), suggesting that the developmental trajectory for monitoring one's own knowledge and ignorance is gradual. However, there is a reason to believe that children have some implicit understanding of the relationship between seeing and knowing in a partial exposure task. Various studies suggest that even before children can explicitly verbalize their knowledge states, they can monitor their uncertainty (e.g., [19, 21, 22]) and display different behaviors concerning items they accurately vs. inaccurately remember or items that are learnable vs. unlearnable ([18, 20, 21]). For example, in an opt-out paradigm, in which children are given the option of declining to answer, they tend to do so when they are uncertain of their answers—as compared to when they are certain (e.g., [18, 19]). Nevertheless, all these studies concern children’s uncertainty about either their memory for the items that they previously learned or were exposed to or their perceptual identification of items presented to them. In one study, Call and Carpenter did demonstrate that 2.5-year-olds have some implicit understanding of the relationship between seeing and knowing in a partial exposure task [34]. However, all prior studies, including the study of Call and Carpenter [34], involved a task in which children’s own uncertainty might directly guide their *own* subsequent behaviors and responses.

We extended this line of research by investigating children’s implicit understanding of a relationship between seeing and knowing in a novel way. We used an informing context in which children’s own uncertainty or ignorance might impact their communication with others. Following the rationale of the opt-out paradigm, we allowed children to decline to inform. We found that children declined to inform another person more often when they did not know than when they knew which object was in a box.

We also examined children’s production of uncertainty gestures, in light of the proposal that gestures communicate speakers’ epistemic states and that children’s gestures might indicate an early, implicit sensitivity to their own ignorance. Children’s gestures are a window to their implicit knowledge of a concept that their verbal responses fail to reveal (e.g., [27]). Moreover, adult listeners understand gestures that mark speakers’ epistemic states as a reliable index for the speakers’ uncertainty [29] and children as young as 7 years old produce uncertainty gestures and are able to detect them in others [30]. The present findings provide some initial evidence suggesting that young children’s uncertainty gestures display an early indication of metacognitive abilities. We found that similar to children’s informing decisions children tended to produce uncertainty gestures more frequently when they did not know the content of a box than when they did know.

Interestingly, children’s decisions to agree (as opposed to refusals) to inform decreased linearly across the three conditions: the less information they had, the less likely they were to agree to inform. Moreover, children’s spontaneous production of uncertainty gestures increased linearly across the three conditions. Therefore, children experienced uncertainty as a function of the total amount of information available to them, rather than as a dichotomous Yes-No judgment, as prompted by the experimenter's explicit question. Feelings of knowing, based on the partial information pertaining to the target that is available to the subject, are not a matter of all or nothing [16]. Cues such as response latency, coherence of incoming information, and difficulty in response selection or production have been shown to reliably guide a decision when they are diagnostic (i.e., when valid and relevant to the task at hand, which seems to be the case in the informing task); when they are not, they generate illusory feelings of knowing [35] or illusory judgments of truth [36].

However, these implicit metacognitive decisions do not seem to correlate with children’s explicit judgments of knowing. In the partial knowledge condition, both children’s decisions to inform and their metacognitive gestures sharply diverge from their verbal responses. Interestingly, at least among 4-year-olds, uncertainty gestures in the Partial knowledge condition were produced as frequently as in the Ignorance condition, both of which differed from the Full knowledge condition. Such a contrast between implicit and explicit metacognitive decisions has been documented in other studies. For example, Paulus et al. found that 3.5-year-old children are able to monitor their memory implicitly (as measured through pupil dilation and fixation on a Smiley scale), but unable to report confidence judgments about the accuracy of their responses [22]. Similarly, young children are able to monitor their cognitive processes by selecting trials depending on difficulty of the trials [18, 20, 21, 31], while unable, at this age, to form reliable verbal reports about what they know (e.g., [13, 19]). A similar contrast has also been found in mindreading studies: infants show an early sensitivity to the false beliefs of others (e.g., [37]), but it is not until the age of 4 or 5 years that children succeed in explicitly reporting a protagonist’s behavior or belief (see [38]).

A common way of framing this contrast consists in emphasizing that language provides children with concepts of mental states (including knowledge and beliefs). Concept-based reasoning might allow children to describe their feelings of confidence in rich inferential terms, and help them guide their decisions (see [39]). However, although the wording of a question may invite a concept-based answer, it is not obvious that the concepts that the children understand and use to address the test question are identical to the experimenter's concepts. With this observation in mind, how can we interpret the contrast in accuracy between implicit and explicit responses?

A first possibility is to maintain that children have the same understanding of the word "know” as the experimenter’s, but have executive difficulties in using the mental concepts in their possession [37]. Alternatively, overattribution of knowledge to themselves in the explicit metacognitive judgments might be related to their desire to contribute actively and communicate more than they actually know (see [40]). It is plausible that such a desire is heightened when reporting one's knowledge in an individualistic context rather than informing (and guiding the action of) someone else.

A second possibility is that children do not understand the word "know” exactly as the experimenter does, and have less differentiated proto-concepts of mental states than adults [41]. Hence, children might have problems in relating their proto-concept of "know" to their experienced uncertainty. A problem with this account, however, is that concept-based metacognition, which presumably depends on mature propositional beliefs, does not functionally replace implicit, experience-based metacognition in adults as it should on this second interpretation. Rather, experience-based metacognition remains an option throughout life [16, 42].

A third possible interpretation is that the contrast between explicit and implicit performance is generated by a duality in the sources of epistemic sensitivity. In the informing task, younger children might have a readily available set of non-conceptual categorical representations associated with opportunities for action [43–46]. In making a verbal report, by contrast, they have to focus on the recently acquired, only partially understood concept of "know” (for further discussion of partially understood concepts: see [47]). The incomplete mastery of this concept would allow children to reliably respond only in the "full knowledge" and the "ignorance" conditions. Future research should address the exact processes respectively involved in implicit and explicit metacognitive judgments and decisions.

Finally, it is worth noting that in our informing task, in contrast with the explicit task, a third person was involved; the collaborative nature of the task plausibly increases children’s motivation for conveying reliable information. As a result, children should tend to monitor more closely what they did or did not perceive, and hence, should be more sensitive to what they actually know than in the explicit task, where their motivation might be related to their own self-concept.

Moreover, our two tasks may differ in their conversational demands. In the informing task, children were asked to tell someone what the object was in the box: the only type of acceptable answer, then, is a specific object identity. In the explicit task, in contrast, children could claim to know without being specific about what they know. This difference in conversational demands might explain the discrepant findings between the two tasks without requiring metacognitive appraisals. However, different conversational demands do not entail that metacognitive appraisals were not involved in fulfilling these demands (see [46]). Granting that questions involving specifications require a deeper processing of *informativeness* than general questions normally do, an ability to monitor informativeness in one's responses—albeit implicitly—should be involved in the informing task. Reporting whether one knows or does not know something, in contrast, does not need to involve in-depth processing of what one knows. Children may rather merely monitor the fluency of the alternative answers to respond: the more *fluently accessible* answer will be one based on the partial access they have had to the object type, ignoring the constraint of specifying object identity.

In general, as discussed earlier, social communicative context may facilitate metacognitive abilities of assessing their own knowledge states. What feature of such contexts (e.g., conversation, helping context in the informing task), however, facilitated children’s performance is open to questions and subject to future research. Additionally, although the explicit task followed the informing task, the enhanced performance in the informing tasks had no effect on the explicit task. Future studies may also address possible effects of the informing task on the explicit task.

The present research adds to the debate about the nature and development of implicit metacognitive abilities and their relationship to concept-based explicit metacognition. Even though they are not definitive, the present findings suggest that young children are sensitive to their own ignorance or uncertainty, a vital skill for being a cooperative social partner, and being able to inform both selectively and reliably.

## Supporting Information

S1 File Data. Data in an excel file.
(XLSX)
